# (−)-Epigallocatechin-3-gallate derivatives combined with cisplatin exhibit synergistic inhibitory effects on non-small-cell lung cancer cells

**DOI:** 10.1186/s12935-019-0981-0

**Published:** 2019-10-14

**Authors:** Jing Wang, Peiyuan Sun, Qi Wang, Pan Zhang, Yuna Wang, Chengting Zi, Xuanjun Wang, Jun Sheng

**Affiliations:** 1 Key Laboratory of Pu-er Tea Science, Ministry of Education, Yunnan Agricultural University, Kunming, Yunnan China; 2 College of Science, Yunnan Agricultural University, Kunming, 650201 Yunnan China; 3 College of Food Science and Technology, Yunnan Agricultural University, Kunming, Yunnan China; 4 State Key Laboratory for Conservation and Utilization of Bio-Resources in Yunnan, Kunming, Yunnan China

**Keywords:** NSCLC, Synthesis, Cisplatin, EGCG derivatives, EGFR

## Abstract

**Background:**

Non-small-cell lung cancer (NSCLC) is the leading cause of cancer-related death worldwide. The inhibition of epidermal growth factor receptor (EGFR) signaling by tyrosine kinase inhibitors or monoclonal antibodies plays a key role in NSCLC treatment. Unfortunately, these treatment strategies are limited by eventual resistance and cell lines with differential EGFR status. Therefore, new therapeutic strategies for NSCLC are urgently required.

**Methods:**

To improve the stability and absorption of (−)-epigallocatechin-3-gallate (EGCG), we synthesized a series of EGCG derivatives. The antitumor activities of EGCG derivatives with or without cisplatin were investigated in vitro and vivo. Cell proliferation, cell cycle distribution and apoptosis were measured in NSCLC cell lines and in vivo in a NCI-H441 xenograft model.

**Results:**

We found that the EGCG derivatives inhibited cell viability and colony formation, caused cell cycle redistribution, and induced apoptosis. More importantly, the combination of the EGCG derivative and cisplatin led to increased growth inhibition, caused cell cycle redistribution, and enhanced the apoptosis rate compared to either compound alone. Consistent with the experiments in vitro, EGCG derivatives plus cisplatin significantly reduced tumor growth.

**Conclusions:**

The combination treatment was found to inhibit the EGFR signaling pathway and decrease the expression of p-EGFR, p-AKT, and p-ERK in vitro and vivo. Our results suggest that compound **3** is a novel potential compound for NSCLC patients.

## Background

Lung cancer is one of the most commonly occurring cancers and the principal cause of cancer-related mortality worldwide [1]. Approximately 85% of lung cancers are non-small-cell lung cancer (NSCLC), including lung adenocarcinoma, squamous cell carcinoma (SCC), and large-cell carcinoma (LCC) histological subtypes, and together these account for more than 1.5 million deaths per year [2, 3]. Approximately 50% of lung cancer patients possess epidermal growth factor receptor (EGFR) overactivated. Recently, major advances in molecular diagnosis and targeted therapies have provided the opportunity to select lung cancer patients, and EGFR has become the new promising target [4, 5].

EGFR is a member of the HER family of receptors, which includes EGFR/HER1/ErbB1, HER2/ErbB2, HER3/ErbB3, and HER4/ErbB4 [6]. EGFR consists of an extracellular module (comprising domains I, II, III, and IV) and an intracellular kinase domain (with a long regulatory C-terminal tail), which are connected by a single-helix transmembrane segment and a juxtamembrane segment [7, 8]. EGFR signaling plays a critical role in regulating the maintenance, differentiation, and growth of epithelial tissues. EGFR signaling activation is frequently observed in lung cancer and EGFR mutations have been observed in many cancer cells, and a high EGFR level is correlated with an advanced stage of the disease and a poor prognosis [9]. Thus, EGFR and its signaling components can be used as targets in the development of new drugs for lung cancer treatment.

**Fig. 1:**
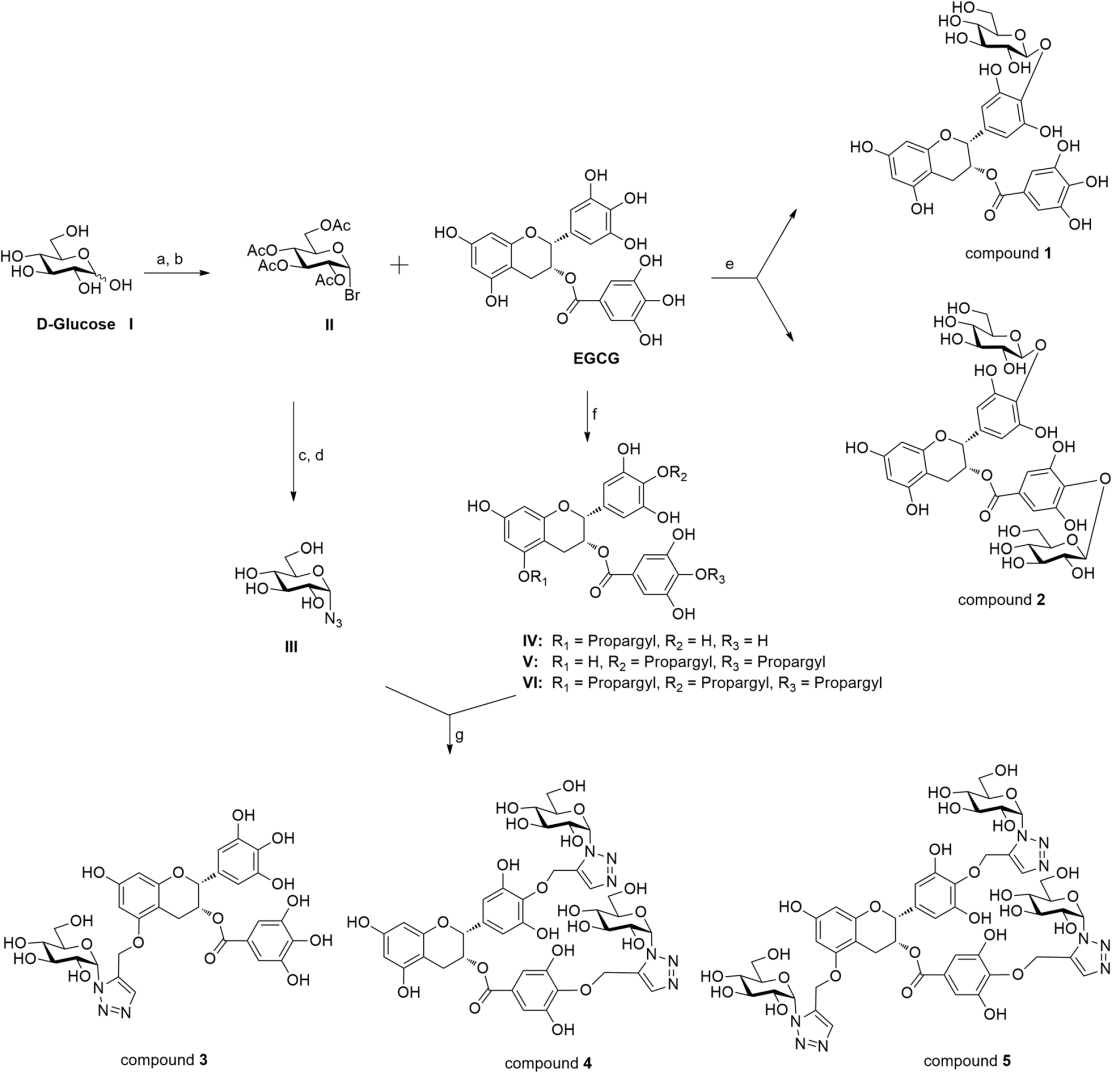
Synthesis of the EGCG derivatives 1–5. Reagents and conditions: **a** Ac_2_O, NaOAc, 100 °C, 20 min, 100%; **b** HBr, AcOH, CH_2_Cl_2_, 0 °C, 96%; **c** NaN_3_, DMF, 90%; **d** CH_3_ONa, CH_3_OH, rt, 78%; **e**, **i** K_2_CO_3_, acetone, 55 °C, 12 h, (ii) CH_3_OH–THF, KOH, 13 h, 0 °C, 11–23%; **f** propargyl bromide, NaH, DMF, 0 °C, 4 h, 11–45%; **g** copper(II) acetate, sodium ascorbate, *t*-BuOH–H_2_O (1:1), THF, rt, 2 h, 56–78%

Green tea is one of the most widely consumed tea beverages around the world [10]. The major catechins in green tea are (−)-epigallocatechin-3-gallate (EGCG), (−)-epigallocatechin (EGC), (−)-epicatechin-3-gallate (ECG), and (−)-epicatechin (EC) (Fig. 1) [11]. EGCG is one of the most abundant and biologically active compound in green tea [11]. There is considerable evidence that EGCG inhibits tumorigenesis, signal transduction pathways, cell invasion, angiogenesis, and metastasis. Moreover, a previous study reported that EGCG also inhibits the activation of EGFR, human epidermal growth factor receptor 2 (HER2), and multiple downstream signaling pathways in cancer cell lines [12]. Liang et al. discovered that EGCG binds to and inhibits the tyrosine kinase activity of EGFR in human A431 epidermoid carcinoma cells [13]. However, EGCG also has several limitations, such as poor stability and membrane permeability [14].

Platinum-based chemotherapy, such as cisplatin (cDDP), is one of the first-line treatments for many types of cancer, including lung cancer [15]. cDDP is a conventional treatment for most advanced NSCLC patients, but it has no significant therapeutic effect [16]. It has been found that EGCG combined with cDDP could strongly inhibit ovarian cancer cell growth [15]. To improve the stability and absorption of EGCG, in this study we synthesized a series of EGCG derivatives, compounds **1**–**5**, and investigated their effects on cell proliferation, clone formation, apoptosis induction, and cell cycle redistribution of NSCLC cell lines either alone or administered together with cDDP.

The results demonstrated that these two agents cause synergistic growth arrest and induce apoptosis. Thus, in the present study, we have evaluated the potential of a therapeutic approach using EGCG derivatives plus cDDP for the treatment of NSCLC and explored the mechanisms of the resulting antitumor efficacy.

## Materials and methods

### Materials

d-Glucose (1) was purchased from Sinopharm Chemical Reagent Co., Ltd. (Shanghai, China). EGCG was obtained from Chengdu Biopurify Phytochemicals, Ltd. (Chengdu, China). 3-(4,5-Dimethylthiazol-2-yl)-2,5-diphenyltetrazolium bromide was obtained from Sigma-Aldrich (MO, USA). Solvents were purified and dried by standard procedures and stored over 4 Å molecular sieves. All other chemicals used were of analytical grade. MS data were obtained in the ESI mode on an API QStar Pulsar instrument. HRMS data were obtained in the ESI mode on an LCMS-IT-TOF instrument (Shimadzu, Kyoto, Japan). NMR spectra were acquired on Bruker AV-400 or DRX-500 instruments (Bruker BioSpin GmbH, Rheinstetten, Germany), using tetramethylsilane (TMS) as the internal standard. Column chromatography was performed on flash silica gel (200–300 mesh; Qingdao Makall Group Co., Ltd., Qingdao, China). All reactions were monitored using thin-layer chromatography (TLC) on silica gel plates.

Dimethylthiazol-2-yl)-2,5-diphenyltetrazolium bromide (MTT) purchased from Sigma-Aldrich (St. Louis, MO, United States). The EGCG derivatives and cDDP were dissolved in dimethyl sulfoxide (DMSO), stored at − 20 °C, and diluted in fresh medium prior to use. The final concentration of DMSO never exceeded 0.1% v/v. Antibodies against phospho-EGFR (Tyr1068), Bax, Bcl-2, PARP-1, caspase-3, caspase-9, and phosphorylated mitogen-activated protein kinase (phospho-MAPK) were obtained from Cell Signaling Technology (Beverly, MA, USA). Antibodies against Akt, phospho-Akt, p44/42 MAPK (Erk1/2), cyclin D1, cyclin E, PCNA, p21, p53, and EGFR were obtained from Abcom (Lake Placid, NY, USA). The anti-β-tubulin antibody was obtained from Proteintech (Rosemont, IL, United States). Anti-mouse IgG peroxidase-linked whole antibodies and anti-rabbit IgG peroxidase-linked species-specific whole antibodies were from Thermo Fisher Scientific (Waltham, MA, United States).

### Synthesis of EGCG derivatives

#### General procedure for the synthesis of the intermediate products IV, V, and VI

A solution of (−)-epigallocatechin-3-gallate (1.4 g, 3.0 mmol) in dry DMF (10 mL) was added to a suspension of sodium hydride (180 mg, 4.5 mmol) at 0 °C under nitrogen. The mixture was stirred at room temperature for 0.5 h, then propargyl bromide (0.2 mL, 3 mmol) was quickly added and the reaction was stirred at 80 °C overnight. After cooling, the mixture was concentrated under vacuum and the resulting residue was purified by silica gel chromatography with CHCl_3_/CH_3_OH, (15:1→9:1) to afford the major product **IV** (682.2 mg, 45%), **V** (440.7 mg, 27%), and VI (192.3 mg, 11%).

#### General procedure for the synthesis of compound 1 and 2

(−)-Epigallocatechin-3-gallate (0.5 g, 1.1 mmol) was dissolved in actetone (10 mL) and d-glucopyranosyl bromide (0.5 g, 1.1 mmol) was added with stirring, potassium carbonate (0.3 g, 2.2 mmol) with heating at 55 °C for 12 h. The mixture was filtered, the filtrate was concentrated and dried in vacuo. Then, the crude product was dissolved in methanol (3 mL) and a potassium hydroxide solution (0.8 mmol, in H_2_O) was added. The mixture was stirred at 0 °C for 72 h and then neutralized with Dowex 50WX4-400 ion-exchange resin to pH ≈ 7. The solvent was evaporated and the residue was purified by column chromatography (CHCl_3_/CH_3_OH/H_2_O, 64%:31%:0.5%) to afford compound **1** (85 mg, 17%) and **2** (101 mg, 20%).

#### General procedure for the synthesis of EGCG derivatives compound 3–5

To a solution of d-glucosyl azide (3) (0.1 mmol/0.2 mmol/0.3 mmol) and **IV**/**V**/**VI** (0.1 mmol) in THF (1.0 mL) and *t*-BuOH–H_2_O (1:1, 1.0 mL) were added copper(II) acetate (0.01 mmol) and sodium ascorbate (1.0 M in H_2_O, 0.1 mL). The reaction mixture was stirred at room temperature for 2 h until disappearance of the starting material as indicated by TLC. The mixture was then evaporated and the residue was purified by column chromatography to afford the cycloaddition products compound **3** (54.7 mg, 78%), compound **4** (60.0 mg, 70%), or compound **5** (66.5 mg, 56%), respectively.

#### Cell cultures

The human NSCLC cell lines NCI-H1975, NCI-H441, and A549, as well as the human colorectal adenocarcinoma cell line Caco-2, were obtained from the American Type Culture Collection (ATCC). The NCI-H1975 cell line contains T790M (exon 20) and L858R (exon 21) point mutations, the NCI-H441 cell lines contains wild-type EGFR and KRAS codon 12 mutant, the A549 cell lines contain the wild-type EGFR. All of the NSCLC cell lines were cultured in RPMI-1640 medium (Thermo Fisher Scientific, Pittsburgh, PA, USA) supplemented with 50 IU/mL of penicillin, 50 mg/L of streptomycin (Solarbio, Beijing, China), and 10% of fetal bovine serum (HyClone, CA, USA) at 37 °C in a humidified 5% CO_2_ incubator. The human colorectal adenocarcinoma cell line Caco-2 was cultured in DMEM high-glucose medium (Thermo Fisher Scientific, Pittsburgh, PA, USA) in the presence of 10% FBS, 50 IU/mL of penicillin, and 50 mg/L of streptomycin.

#### Cell proliferation

The effects of the EGCG derivatives on the survival of NSCLC cells were determined using the MTT assay. A549, NCI-H441, and NCI-H1975 cells were seeded in 96-well plates (3 × 10^4^ cells/well) and then treated with one of the EGCG derivatives alone (0, 30, 60, 90, 120, or 150 µM), cDDP alone (12 µM), or 12 μM cDDP plus one of the EGCG derivatives (100 µM) for 48 h. MTT was then added to the cells and the plates were incubated for a further 4 h. After removal of the culture medium, the produced MTT formazan crystals were dissolved with 150 µL DMSO and measured at 492 nm using a microplate reader. The percentage of inhibition was calculated as follows: inhibition ratio (IR, %) = (1 − OD(sample)/OD(control)) × 100%. The experiments were carried out in triplicate, and the IC_50_ (the concentration of drug that inhibits cell growth by 50%) values were determined.

#### Colony formation

NCI-H441 cells (5 × 10^3^ cells per well) were seeded in six-well plates and treated with the indicated agents for 48 h. The cells were then trypsinized and 1000 single viable cells were plated in each well of a six-well plate. The cells were cultured for an additional 14 days then stained with 0.5 mL of 0.005% crystal violet solution for at least 0.5 h, and the colonies were counted under a light microscope.

#### Cell apoptosis assay

Cell apoptosis was quantified using an annexin V/propidium iodide (PI) detection kit (BD Biosciences, PA, USA) and flow cytometry. Cells (5 × 10^5^/well) were plated in six-well dishes and then treated with the EGCG derivatives with or without cDDP. After the treatment, the collected cells were incubated in 100 µL of binding buffer and 5 µL of FITC annexin V and 10 µL of PI were added to the suspension. The mixtures were gently vortexed and then incubated for 15 min at room temperature in the dark before performing the flow cytometry measurements (BD FACSCalibur) within 1 h.

#### Cell cycle analysis by propidium iodide (PI) staining

Aliquots of 5 × 10^5^ cells were harvested, washed with phosphate-buffered saline (PBS), and then fixed in 70% ethanol at 4 °C overnight. After fixation, the cells were washed twice with cold PBS and stained in binding buffer containing 1 µg/mL of propidium iodide (BD Biosciences) and RNase for 0.5 h. The samples were then analyzed by flow cytometry (BD FACSCalibur).

#### Western blotting

Samples containing equal amounts of proteins as indicated in the text were resolved by SDS polyacrylamide gel electrophoresis and transferred to PVDF membranes. The membranes were blocked with 5% BSA at room temperature for 1 h and then probed with primary antibodies overnight at 4 °C and incubated with the HRP-conjugated secondary antibodies for 1 h at room temperature. HRP was detected using the Pro-light HRP Chemiluminescent Kit (Tiangen Biotech, Beijing, China) and FluorChem E System (ProteinSimple, Santa Clara, CA, United States).

#### Transport studies

Caco-2 cells were grown as epithelial monolayers, seeded onto fibrillar collagen-coated polyethylene terephthalate (PET) Corning transwell inserts (1.12 cm^2^) with a pore size of 3 μm at a density of 2.0 × 10^5^ cells/insert, and incubated at 37 °C in an atmosphere of 5% CO_2_ and 95% relative humidity in complete DMEM. The medium was replaced every 2 days with 0.5 mL medium in the apical (AP) side and 1.5 mL in the basolateral (BL) side over a period of 21 days. The integrity of the cell monolayer was determined by measuring the TEER (transepithelial electrical resistance) using an epithelial voltohmmeter (EVOM). The TEER values for each monolayer were measured every 2 days until they exceeded 350 Ω cm^2^. To ensure the integrity of the Caco-2 monolayers, the TEER values were monitored before and after the experiments.

To measure the transport of EGCG and compound **3** across the Caco-2 cell monolayer, both sides of the transwell were equilibrated for 30 min with warm Hank’s balanced salt solution (HBSS). The transport buffer containing EGCG and compound **3** was added to either the apical (0.5 mL) or the basolateral (1.5 mL) side of the insert, while the receiving compartment contained the corresponding volume of transport buffer. After incubation for 30 or 60 min, 100 μL samples were taken from the receiving chamber and immediately replenished with an equal volume of pre-warmed HBSS. The samples were acidified with an equal volume of 5% formic acid and then frozen until LC–MS analysis.

The apparent permeability coefficients (*P*_app_) of the compounds were calculated using the following equation:

$$P_{\text{app}} = \frac{{\text{dQ}}}{{\text{dt}}} \times \frac{1}{{A \cdot C_{0} }},$$

where *dQ*/*dt* is the amount of solute transported across the Caco-2 barrier in time *dt*, *C*_0_ is the solute concentration in the apical compartment at time zero, and *A* is the cross-sectional area of the epithelium in contact with the apical solution.

#### In vivo animal study

Twenty-four male BALB-C/nude mice were purchased from the Cavens Lab Animal (Changzhou, China). The mice were subcutaneously injected in the dorsal with NCI-H441 cells (4 × 10^6^/0.2 mL PBS per mouse). The mice were randomly divided into four groups, including control, compound **3**, cDDP, and compound **3** plus cDDP (n = 6, per group). The mice were treated with compound **3** (200 mg/kg per day) and cDDP (2 mg/kg per 2 days) alone or in combination. All drugs were given through intra-peritoneal injection. Tumour size was measured once every 2 days with a calipre (volume mm^3^ = length × width × width/2). Body weight was recorded once every 2 days. After there weeks of treatment, all mice were euthanized by cervical dislocation and the tumors were excised into formalin, and portion of the tumor fresh frozen in liquid nitrogen for further processing and analysis.

#### Immunohistochemistry staining

Three micrometer sections were cut, deparaffinized in xylenes, rehydrated in ethanol, and washed in phosphate-buffered saline. The antigen was retrieved at 95 °C for 20 min in 0.01 M citrate buffer (pH 6.0). Then incubated with primary antibodies overnight at 4 °C, followed by incubation with the secondary antibody at RT for 1 h. The immunostaining was performed using DAB, and counterstained with hematoxylin.

#### Docking studies

The X-ray crystal structure of EGFR (PDB code: 2ITY) was retrieved from protein data bank (http://www.pdb.org). AutoDock Tools v1.56 was used to perform the molecular docking experiment according to the literature [17]. Docking parameters were set the default values, except number of GA runs was set to 20 and maximum number of evals (medium) was set to 5,000,000. The lowest binding energy conformers were selected out of 10 different conformers for each docking simulation and resultant data was further analyzed.

#### Statistical analysis

All results are expressed as the mean ± the standard deviation (SD) from three or more independent replicates. The data were statistically analyzed with either the Student’s t test or one-way ANOVA. p < 0.05 was considered statistically significant. All of the statistical analyses were performed using the GraphPad Prism 5.0 software (San Diego, CA, USA).

## Results

### Chemistry

The EGCG derivatives (compounds **1**–**5**) were prepared according to Fig. 1. Compound **1** and **2** were prepared in 11–27% yield by the treatment of d-glucopyranosyl bromide (2) [18] and EGCG with potassium carbonate and potassium hydroxide solution (0.5 M in CH_3_OH) as previously described [19]. The preparation of **IV**, **V** and **VI** were obtained as the major products in 11–45% yield by the reaction of EGCG with sodium hydride and propargyl bromide using a similar method as reported in the literature. To introduce the azido functionality for the click reaction, d-glucose was readily converted to d-glucosyl azide (3) according to known procedures [18]. d-Glucosyl azide (3) was then reacted with the alkynes **IV**, **V** and **VI** in the presence of copper (II) acetate and sodium ascorbate in *tert*-butyl alcohol and water (1:1) at room temperature for 2 h to afford the EGCG glycosides compound **3**–**5** in 56–78% yield. The derivatives were characterized by ^1^H-NMR, ^13^C-NMR, ESI–MS, and HRESI-MS, which were consistent with the proposed structures.

#### *(2R,3R)-7-Hydroxy-5-(prop-2-yn-1-yloxy)-2-(3,4,5-trihydroxyphenyl)chroman-3-yl 3,4,5-trihydroxybenzoate* (**IV**)

Yield: 45%, ^1^H-NMR (CD_3_OD, 500 MHz) *δ* 6.90 (s, 2H, C^2″^-H, C^6″^-H), 6.50 (s, 2H, C^2′^-H, C^6′^-H), 5.96 (s, 2H, C^6^-H, C^8^-H), 5.53 (brs, 1H, C^3^-H), 4.97 (s, 1H, C^2^-H), 4.78 (d, 2H, *J* = 2.4 Hz, OCH_2_), 3.29 (t, 1H, *J* = 1.6 Hz, C≡CH), 2.96 (dd, 1H, *J* = 4.6 Hz, 12.0 Hz, C^4^-H_a_), 2.85 (dd, 1H, *J* = 4.6 Hz, 12.0 Hz, C^4^-H_b_); ^13^C-NMR (CD_3_OD, 125 MHz) *δ* 167.0 (C=O), 157.9 (C-7), 157.8 (C-9), 157.2 (C-5), 151.9 (C-3^′^, C-5^′^), 146.7 (C-3^″^, C-5^″^), 138.4 (C-4^″^), 133.8 (C-4^′^), 130.7 (C-1^′^), 127.1 (C-1^″^), 110.1 (C-2^″^, C-6^″^), 106.8 (C-2^′^, C-6^′^), 99.3 (C-10), 96.5 (C-8), 95.9 (C-6), 80.4 (C-2), 79.5 (*C*≡CH), 78.5 (C≡*C*H), 76.7 (C-3), 60.0 (OCH_2_), 26.8 (C-4); ESIMS: *m/z* 495 [M−H]^–^.

#### *(2R,3R)-2-(3,5-Dihydroxy-4-(prop-2-yn-1-yloxy)phenyl)-5,7-dihydroxychroman-3-yl 3,5-dihydroxy-4-(prop-2-yn-1-yloxy)benzoate* (**V**)

Yield: 27%, ^1^H-NMR (CD_3_OD, 500 MHz) *δ* 6.90 (s, 2H, C^2″^-H, C^6″^-H), 6.52 (s, 2H, C^2′^-H, C^6′^-H), 5.96 (s, 2H, C^6^-H, C^8^-H), 5.56 (brs, 1H, C^3^-H), 5.00 (s, 2H, C^2^-H), 4.77 (d, 2H, *J* = 2.4 Hz, OCH_2_), 4.67 (d, 2H, *J* = 2.4 Hz, OCH_2_), 3.30 (t, 2H, *J* = 1.6 Hz, 2 × C≡CH), 2.98 (dd, 1H, *J* = 4.6 Hz, 12.1 Hz, C^4^-H_a_), 2.86 (dd, 1H, *J* = 4.6 Hz, 12.1 Hz, C^4^-H_b_); ^13^C-NMR (CD_3_OD, 125 MHz) *δ* 167.0 (C=O), 157.9 (C-7), 157.8 (C-9), 157.0 (C-5), 151.9 (C-3^′^, C-5^′^), 151.8 (C-3^″^, C-5^″^), 139.4 (C-4^″^), 136.3 (C-4^′^), 134.6 (C-1^′^), 127.0 (C-1^″^), 110.1 (C-2^″^, C-6^″^), 106.8 (C-2^′^, C-6^′^), 99.3 (C-10), 96.6 (C-8), 95.9 (C-6), 80.4 (C-2), 80.0 (*C*≡CH), 78.2 (*C*≡CH), 76.7 (C≡*C*H), 76.4 (C≡*C*H), 70.2 (C-3), 60.2 (OCH_2_), 60.0 (OCH_2_), 26.8 (C-4); ESIMS: *m/z* 533 [M−H]^−^.

#### *(2R,3R)-2-(3,5-Dihydroxy-4-(prop-2-yn-1-yloxy)phenyl)-7-hydroxy-5-(prop-2-yn-1-yloxy)chroman-3-yl 3,5-dihydroxy-4-(prop-2-yn-1-yloxy)benzoate* (**VI**)

Yield: 11%, ^1^H-NMR (CD_3_OD, 500 MHz) *δ* 6.88 (s, 2H, C^2″^-H, C^6″^-H), 6.52 (s, 2H, C^2′^-H, C^6′^-H), 6.00 (s, 2H, C^6^-H, C^8^-H), 5.75 (brs, 1H, C^3^-H), 5.12 (s, 1H, C^2^-H), 4.79 (d, 6H, *J* = 2.4 Hz, 3 × OCH_2_), 3.29 (t, 3H, *J* = 1.6 Hz, 3 × C≡CH), 2.98 (dd, 1H, *J* = 4.6 Hz, 12.0 Hz, C^4^-H_a_), 2.85 (dd, 1H, *J* = 4.6 Hz, 12.0 Hz, C^4^-H_b_); ^13^C-NMR (CD_3_OD, 125 MHz) *δ* 166.7 (C=O), 158.2 (C-7), 158.0 (C-9), 156.2 (C-5), 152.1 (C-3^′^, C-5^′^), 151.8 (C-3^″^, C-5^″^), 149.2 (C-4^″^), 139.3 (C-4^′^), 126.2 (C-1^′^), 117.7 (C-1^″^), 110.4 (C-2^″^, C-6^″^), 110.0 (C-2^′^, C-6^′^), 98.9 (C-10), 97.2 (C-8), 95.7 (C-6), 80.0 (C-2), 77.4 (*C*≡CH), 76.7 (*C*≡CH), 76.7 (*C*≡CH), 76.7 (C≡*C*H), 76.7 (C≡*C*H), 76.3 (C≡*C*H), 68.2 (C-3), 60.0 (OCH_2_), 54.5 (OCH_2_), 23.7 (C-4); ESIMS: *m/z* 571 [M−H]^−^.

#### *(2R,3R)-5,7-Dihydroxy-2-(3,4,5-trihydroxyphenyl)chroman-3-yl 3,5-dihydroxy-4-(((2S,3R,4S,5S,6R)-3,4,5-trihydroxy-6-(hydroxymethyl)tetrahydro-2H-pyran-2-yl)oxy)benzoate* (compound **1**)

Yield: 17%, ^1^H-NMR (CD_3_OD, 500 MHz) *δ* 6.82 (s, 2H, C^2″^-H, C^6″^-H), 6.52 (s, 2H, C^2′^-H, C^6′^-H), 5.84 (d, 1H, *J* = 2.4 Hz, C^6^-H), 5.84 (d, 1H, *J* = 2.4 Hz, C^8^-H), 5.37 (brs, 1H, C^3^-H), 5.25–5.24 (m, 1H, C^3*‴*^-H), 5.03 (s, 1H, C^2^-H), 4.87 (d, 1H, *J* = 9.0 Hz, C^1*‴*^-H), 4.59-4.57 (m, 1H, C^5*‴*^-H), 3.60–3.58 (m, 2H, C^6*‴*^-H), 3.33–3.15 (m, 2H, C^2*‴*^-H, C^4*‴*^-H), 2.89–2.69 (m, 1H, C^4^-H_a_), 2.58–2.49 (m, 1H, C^4^-H_b_); ^13^C-NMR (CD_3_OD, 125 MHz) *δ* 164.7 (C=O), 156.7 (C-5), 156.5 (C-9), 155.3 (C-7), 150.3 (C-3^′^, 5^*′*^), 149.8 (C-3^*″*^, 5^*″*^), 137.1 (C-4^*″*^), 135.6 (C-4^*′*^), 132.6 (C-1^*″*^), 125.6 (C-1^*′*^), 108.6 (C-2^*″*^, 6^*″*^), 106.0 (C-1^*‴*^), 105.6 (C-2^*′*^, 6^*′*^), 95.7 (C-6), 94.2 (C-8), 92.0 (C-10), 77.3 (C-2), 77.2 (C-5^*‴*^), 76.4 (C-3^*‴*^), 75.1 (C-2^*‴*^), 73.6 (C-4^*‴*^), 69.3 (C-3), 60.4 (C-6^*‴*^); ESIMS: *m/z* 621 [M+H]^+^, HRESIMS: calcd for C_28_H_28_O_16_ [M−H]^−^ 619.1305, found 619.1295.

#### *(2R,3R)-2-(3,5-Dihydroxy-4-(((2S,3R,4S,5S,6R)-3,4,5-trihydroxy-6-(hydroxymethyl)tetrahydro-2H-pyran-2-yl)oxy)phenyl)-5,7-dihydroxychroman-3-yl 3,5-dihydroxy-4-(((2S,3R,4S,5S,6R)-3,4,5-trihydroxy-6-(hydroxymethyl)tetrahydro-2H-pyran-2-yl)oxy)benzoate* (compound **2**)

Yield: 20%, ^1^H-NMR (CD_3_OD, 500 MHz) *δ* 6.80 (s, 2H, C^2*″*^-H, C^6*″*^-H), 6.51 (s, 2H, C^2*′*^-H, C^6*′*^-H), 5.94 (d, 1H, *J* = 2.4 Hz, C^6^-H), 5.84 (d, 1H, *J* = 2.4 Hz, C^8^-H), 5.38 (brs, 1H, C^3^-H), 5.24–5.23 (m, 1H, C^3*‴*^-H), 5.14–5.13 (m, 1H, C^3*‴*^-H), 5.03 (s, 1H, C^2^-H), 4.70 (d, 1H, *J* = 9.0 Hz, C^1*‴*^-H), 4.61–4.60 (m, 1H, C^5*‴′*^-H), 4.56–4.55 (m, 1H, C^5*‴*^-H), 4.46 (d, 1H, *J* = 9.0 Hz, C^1*‴′*^-H), 3.61–3.57 (m, 4H, C^6*‴*^-H, C^6*‴′*^-H), 3.30–3.22 (m, 4H), 2.99–2.89 (m, 1H, C^4^-H_a_), 2.70–2.66 (m, 1H, C^4^-H_b_); ^13^C-NMR (CD_3_OD, 125 MHz) *δ* 164.7 (C=O), 156.7 (C-5), 156.5 (C-9), 155.3 (C-7), 150.3 (C-3^*′*^, 5^*′*^), 149.8 (C-3^*″*^, 5^*″*^), 137.1 (C-4^*″*^), 135.6 (C-4^*′*^), 132.6 (C-1^*″*^), 125.6 (C-1^*′*^), 108.6 (C-2^*″*^, 6^*″*^), 106.0 (C-1^*‴*^), 105.8 (C-2^*′*^, 6^*′*^), 104.6 (C-1^*‴*^), 95.7 (C-6), 94.3 (C-8), 92.1 (C-10), 77.2 (C-2), 77.1, 73.6, 76.1, 75.8, 75.7, 73.6, 69.4 (C-3), 61.2, 60.4, 25.6 (C-4); ESIMS: *m/z* 782 [M+H]^+^, HRESIMS: calcd for C_34_H_38_O_21_ [M−H]^−^ 781.1833, found 781.1814.

#### *(2R,3R)-7-Hydroxy-5-((1-((2R,3R,4S,5S,6R)-3,4,5-trihydroxy-6-(hydroxymethyl)tetrahydro-2H-pyran-2-yl)-1H-1,2,3-triazol-5-yl)methoxy)-2-(3,4,5-trihydroxyphenyl)chroman-3-yl 3,4,5-trihydroxybenzoate* (compound **3**)

Yield: 78%, ^1^H-NMR (CD_3_OD, 500 MHz) *δ* 8.11 (s, 1H, CH–N), 6.91 (s, 2H, C^2*″*^-H, C^6*″*^-H), 6.53 (s, 1H, C^2′^-H), 6.52 (s, 1H, C^6′^-H), 5.96 (s, 1H, C^6^-H), 5.95 (s, 1H, C^8^-H), 5.58 (d, 1H, *J* = 7.6 Hz, C^1*‴*^-H), 5.21 (s, 1H, C^3^-H), 5.11 (s, 1H, C^2^-H), 4.78 (d, 2H, *J* = 2.4 Hz, OCH_2_), 3.89–3.86 (m, 2H), 3.72–3.69 (m, 1H), 3.57–3.53 (m, 2H), 3.51–3.48 (m, 1H), 2.99–2.87 (m, 1H, C^4^-H_a_), 2.85–2.82 (m, 1H, C^4^-H_b_); ^13^C-NMR (CD_3_OD, 125 MHz) *δ* 167.1 (C=O), 157.9 (C-5), 157.8 (C-7), 157.0 (C-9), 151.9 (C-3^*″*^), 151.9 (C-5^*″*^), 151.7 (C-3^*′*^), 151.6 (C-5^*′*^), 146.7 (CH=N), 139.4 (C-4^*″*^), 136.3 (C-4^*′*^), 127.0 (C-1^*′*^), 124.7 (C-1^*″*^), 110.4 (C-2^*″*^), 110.1 (C-6^*″*^), 106.9 (C-2^*′*^), 106.8 (C-6^*′*^), 99.3 (C-10), 96.6 (C-1^*‴*^), 95.9 (C-6), 89.6 (C-8), 81.1 (C-2), 78.4, 76.8, 74.1, 70.8, 70.2 (OCH_2_), 66.0 (C-3), 62.4 (C-6^*‴*^), 26.7 (C-4); ESIMS: *m/z* 702 [M+H]^+^, HRESIMS: calcd for C_31_H_31_N_3_O_16_H [M−H]^−^ 700.1704, found 701.1636.

#### *(2R,3R)-2-(3,5-Dihydroxy-4-((1-((2R,3R,4S,5S,6R)-3,4,5-trihydroxy-6-(hydroxymethyl)tetrahydro-2H-pyran-2-yl)-1H-1,2,3-triazol-5-yl)methoxy)phenyl)-5,7-dihydroxychroman-3-yl3,5-dihydroxy-4-((1-((2R,3R,4S,5S,6R)-3,4,5-trihydroxy-6-(hydroxymethyl)tetrahydro-2H-pyran-2-yl)-1H-1,2,3-triazol-5-yl)methoxy)benzoate* (compound **4**)

Yield: 70%, ^1^H-NMR (CD_3_OD, 500 MHz) *δ* 8.18 (s, 1H, CH–N), 8.12 (s, 1H, CH–N), 6.92 (s, 2H, C^2*″*^-H, C^6*″*^-H), 6.53 (s, 2H, C^2*′*^-H, C^6*′*^-H), 5.96 (s, 2H, C^6^-H, C^8^-H), 5.58 (d, 1H, *J* = 7.6 Hz, C^1*‴*^-H), 5.57 (d, 1H, *J* = 7.6 Hz, C^1*‴′*^-H), 5.22 (C^3^-H), 5.11 (C^2^-H), 5.00 (d, 2H, *J* = 2.4 Hz, 2 × OCH_3_), 3.91–3.85 (m, 4H), 3.71–3.68 (m, 2H), 3.57–3.48 (m, 6H), 2.97 (dd, 1H, *J* = 4.6 Hz, 12.0 Hz, C^4^-H_a_), 2.94 (dd, 1H, *J* = 4.6 Hz, 12.0 Hz, C^4^-H_b_); ^13^C-NMR (CD_3_OD, 125 MHz) *δ* 167.0 (C=O), 157.9 (C-7), 157.8 (C-9), 157.0 (C-5), 151.7 (C-3^′^, C-5^′^), 151.6 (C-3^*″*^, C-5^*″*^), 139.4 (C-4^*″*^), 136.3 (C-4^*′*^), 134.6 (C-1^*′*^), 127.0 (C-1^*″*^), 110.4 (C-2^*″*^, C-6^*″*^), 107.2 (C-2^′^, C-6^′^), 99.2 (C-10), 96.6 (C-8), 95.9 (C-6), 89.5 (C-1^*‴*^), 89.5 (C-1^*‴′*^), 81.1, 78.4, 78.3, 74.1, 74.0, 70.8, 70.8, 70.2 (OCH_2_), 66.0 (C-3), 62.4 (C-6^*‴*^), 62.4 (C-6^*‴′′*^), 26.8 (C-4); ESIMS: *m/z* 945 [M+H]^+^, HRESIMS: calcd for C_40_H_44_N_6_O_21_H [M−H]^−^ 943.2487, found 944.2460.

#### *(2R,3R)-2-(3,5-Dihydroxy-4-((1-((2R,3R,4S,5S,6R)-3,4,5-trihydroxy-6-(hydroxymethyl)tetrahydro-2H-pyran-2-yl)-1H-1,2,3-triazol-5-yl)methoxy)phenyl)-7-hydroxy-5-((1-((2R,3R,4S,5S,6R)-3,4,5-trihydroxy-6-(hydroxymethyl)tetrahydro-2H-pyran-2-yl)-1H-1,2,3-triazol-5-yl)methoxy)chroman-3-yl 3,5-dihydroxy-4-((1-((2R,3R,4S,5S,6R)-3,4,5-trihydroxy-6-(hydroxymethyl)tetrahydro-2H-pyran-2-yl)-1H-1,2,3-triazol-5-yl)methoxy)benzoate* (compound **5**)

Yield: 56%, ^1^H-NMR (CD_3_OD, 500 MHz) *δ* 8.19 (s, 1H, CH–N), 8.13 (s, 1H, CH–N), 8.09 (s, 1H, CH–N), 6.98 (s, 2H, C^2*″*^-H, C^6*″*^-H), 6.57 (s, 2H, C^2*′*^-H, C^6*′*^-H), 5.97 (s, 2H, C^6^-H, C^8^-H), 5.64 (d, 1H, *J* = 7.6 Hz, C^1*‴*^-H), 5.59 (d, 1H, *J* = 7.6 Hz, C^1*‴′*^-H), 5.57 (d, 1H, *J* = 7.6 Hz, C^1*‴′′*^-H), 5.13 (C^3^-H), 5.12 (C^2^-H), 5.05 (d, 2H, *J* = 2.4 Hz, 3 × OCH_3_), 4.09–4.08 (m, 2H), 3.89–3.88 (m, 2H), 3.87–3.85 (m, 4H), 3.72–3.71 (m, 2H), 3.60–3.54 (m, 6H), 2.96 (dd, 1H, *J* = 4.6 Hz, 12.0 Hz, C^4^-H_a_), 2.93 (dd, 1H, *J* = 4.6 Hz, 12.0 Hz, C^4^-H_b_); ^13^C-NMR (CD_3_OD, 125 MHz) *δ* 166.9 (C=O), 158.0 (C-7), 157.9 (C-9), 156.9 (C-5), 151.9 (C-3^′^, C-5^′^), 151.7 (C-3^*″*^, C-5^*″*^), 140.7 (C-4^*″*^), 136.4 (C-4^*′*^), 134.8 (C-1^*′*^), 127.1 (C-1^*″*^), 112.7 (C-2^*″*^, C-6^*″*^), 107.4 (C-2^′^, C-6^′^), 99.1 (C-10), 96.3 (C-8), 95.8 (C-6), 89.6 (C-1^*‴*^), 89.6 (C-1^*‴″*^), 89.6 (C-1^*‴″″*^), 81.1, 78.4, 78.4, 78.1, 74.1, 74.0, 73.8, 70.8, 70.8 (OCH_2_), 66.2 (C-3), 62.4 (C-6^*‴*^), 62.3 (C-6^*‴″*^), 62.3 (C-6^*‴″*^), 26.4 (C-4); ESIMS: *m/z* 1186 [M−H]^+^, HRESIMS: calcd for C_49_H_57_N_9_O_26_H [M−H]^−^ 1186.3342, found 1186.3376.

### Effects of the EGCG derivatives on human NSCLC cell lines

**Fig. 2:**
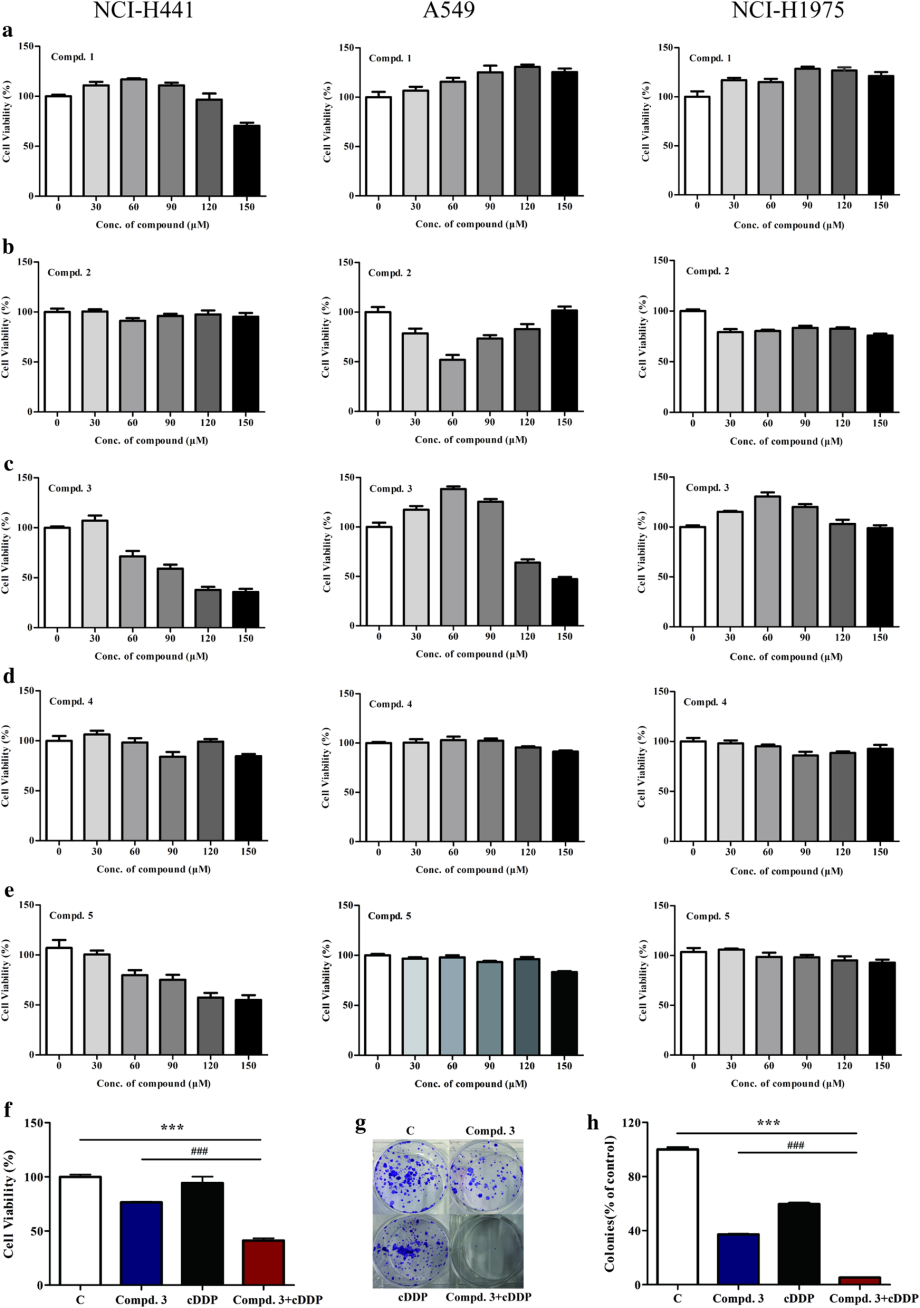
Inhibitory effects of the EGCG derivatives on NSCLC cells. **a**–**e** The inhibitory effects of compounds **1**–**5** on NCI-H441 (left panel), A549 (middle panel), and H1975 (right panel) cells, as evaluated by the MTT assay. **f** NCI-H441 cells were treated with compound **3** (100 μM) and cDDP (12 μM) in combination or individually, prior to the MTT assay. **g**, **h** Clonogenic assay of NCI-H441 cells treated with compound **3** (100 μM), cDDP (12 μM), or both. Data represent the average of three independent experiments (mean ± SD, *p* < 0.001). ***p < 0.001, cDDP + compound **3** vs cDDP; ^###^*p* < 0.001, cDDP + compound **3** vs compound **3**

To investigate the effects of the EGCG derivatives on human NSCLC cell lines, we used three cell lines with different genetic EGFR statuses: A549 (wild-type EGFR), NCI-H441 (wild-type EGFR and KRAS codon 12 mutant), and NCI-H1975 (T790M and L858R point mutations). We first subjected the NSCLC cell lines to various doses of the EGCG derivatives. We observed that proliferation of the NCI-H441 cells was inhibited by increasing doses of compound **3**, with substantial dose-dependent growth inhibition, but the same effect was not observed in the A549 or NCI-H1975 cells (Fig. 2c).

### Combination treatment with compound 3 and cDDP inhibited the growth of NCI-H441 cells

To determine the effect on cell proliferation in NCI-H441 cells, we first conducted a 3-(4,5-dimethylthiazol-2-yl)-2,5-diphenyltetrazolium bromide (MTT) assay after treating the NCI-H441 cells with compound **3** plus cDDP, compound **3** alone, or cDDP alone. As illustrated in Fig. 2f, the inhibition rates for compound **3** combined with cDDP were significantly higher than those for either compound **3** or cDDP alone (*p* < 0.001). These results were further confirmed using a clonogenic assay. Anchorage-independent colony formation assays further proved the synergistic effect on cell proliferation. The results showed that co-treatment with compound **3** and cDDP significantly inhibited NCI-H441 colony formation compared with either compound **3** or cDDP alone (Fig. 2g, h).

### Effect of compound 3 in combination with cDDP on the cell cycle distribution in NCI-H441 cells

**Fig. 3:**
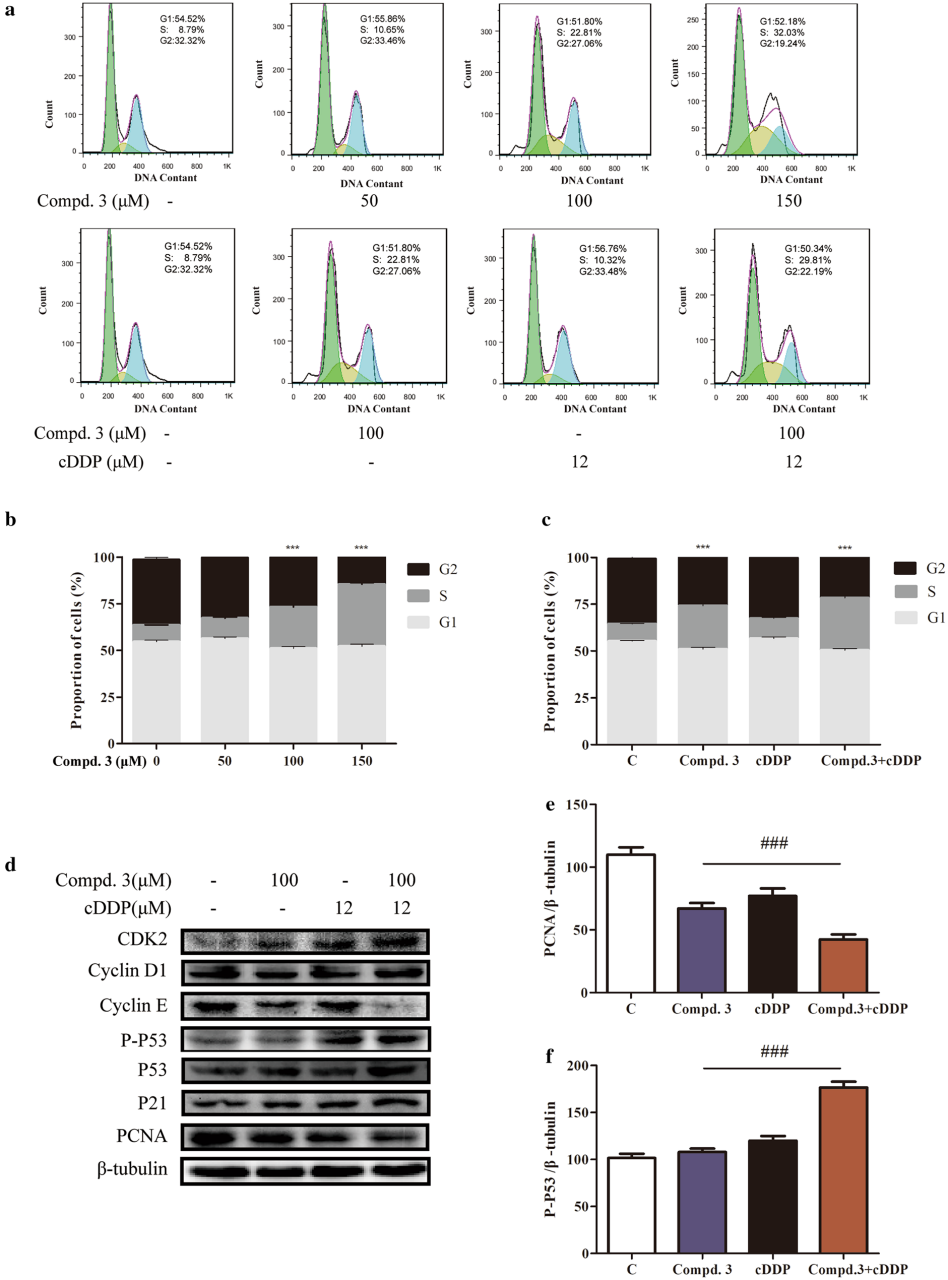
Effects of compound 3, cDDP, or a combination of the two drugs on the cell cycle distribution and proteins associated with cell cycle control in NCI-H441 cells. **a** The cell cycle distribution (sub-G1, G0/G1, S, and G2/M) was determined by flow cytometry. NCI-H441 cells were treated with various concentrations of compound **3** (0, 50, 100, or 150 μM) or treated with compound **3** and cDDP in combination or individually. **b**, **c** The proportions of cells in each phase are expressed as percentages. **d** The levels of cyclin D1, cyclin E, p53, p21, and PCNA were determined by western blotting with β-tubulin as the loading control. **e**, **f** Quantification of relative PCNA and p-p53 protein levels. Data represent the average of three independent experiments (mean ± SD). ***p < 0.001 vs the control; ^###^p < 0.001, cDDP + compound 3 vs compound 3

To further determine why the combination of compound **3** and cDDP caused synergistic inhibition of cell growth, we investigated the cell cycle distribution. The cells were treated with compound **3**, cDDP, or both, and then stained with propidium iodide (PI) and analyzed by flow cytometry. Increasing doses of compound **3** arrested cells in the G1 phase (sub- and G0/G1) and increased the proportion of cells in the S phase in a dose-dependent manner (Fig. 3a, b). Furthermore, in the NCI-H441 cells, the synergistic combination treatment led to an increased proportion of cells in the S phase and a decreased proportion of cells in the sub-G1 or G2/M phases compared to treatment with cDDP alone (*p* < 0.001) (Fig. 3a, c). Thus, compound **3** plus cDDP inhibited cell proliferation by increasing the cDDP-induced S-phase cell cycle arrest.

We further evaluated the effects of the drug combination by examining the levels of proliferating cell nuclear antigen (PCNA), p53, p21, cyclin D1, and cyclin E, each of which have established roles in cell cycle regulation. Treatment with cDDP alone had minimal effects on the levels of these proteins in the NCI-H441 cells. However, the adiministered together with cDDP and compound **3** led to decreased PCNA expression (Fig. 3d, e). These results confirm that the synergistic combination of compound **3** and cDDP enhances cell cycle arrest.

### Combination treatment with compound 3 and cDDP induced apoptosis in NCI-H441 cells

**Fig. 4:**
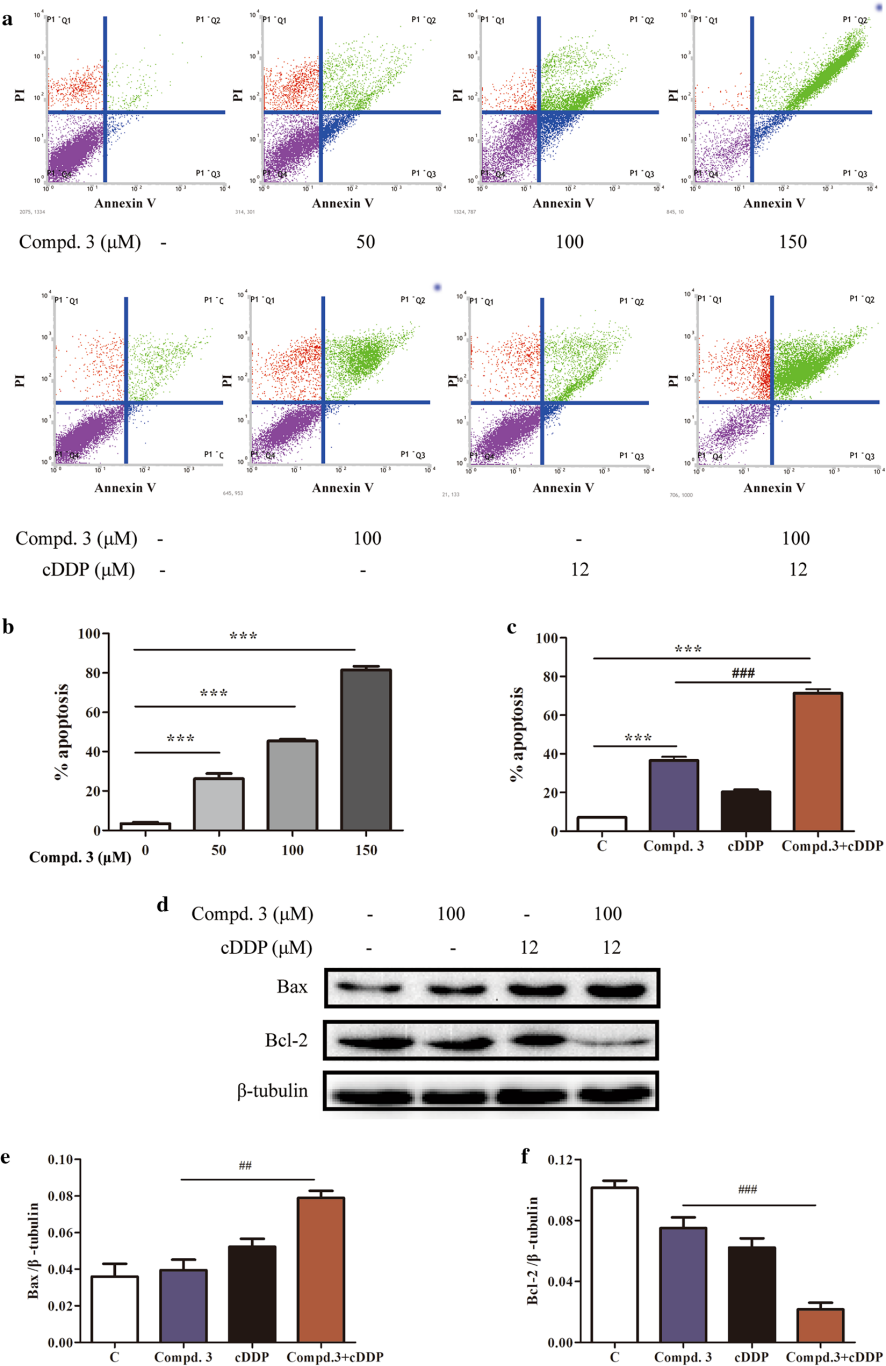
Effects of compound 3, cDDP, or a combination of the two drugs on the induction of NSCLC cell apoptosis and the expression of relevant proteins in NCI-H441 cells. **a** Flow cytometry was used to detect cell apoptosis in NCI-H441 cells treated with various concentrations of compound **3** and cDDP in combination or individually. **b**, **c** The ratio of apoptotic cells in each group are expressed as percentages. **d** The expression levels of Bax and Bcl-2 were determined by western blotting after 12 h of drug treatment, using β-tubulin as the loading control. **e**, **f** Quantification of relative Bax and Bcl-2 protein levels. Data represent the average of three independent experiments (mean ± SD). Data represent the average of three independent experiments (mean ± SD). ***p < 0.001 vs the control; ^###^p < 0.001, ^##^*p* < 0.01, cDDP + compound **3** vs compound **3**

We next determined the type of cell death and apoptosis induced by the combination treatment in NCI-H441 cells. As such, NCI-H441 cells were treated with compound **3**, cDDP, or both for 48 h, subjected to annexin V-FITC/PI staining, and subsequently analyzed by flow cytometry. As shown in Fig. 4a, b, compared to the untreated control, the number of apoptotic cells was significantly increased in a concentration-dependent manner with respect to compound **3**. Furthermore, the synergistic combination treatment led to a significantly increased degree of apoptosis compared to the untreated control and either compound **3** or cDDP alone (Fig. 4a, c).

We also determined the expression levels of Bax and Bcl-2, which are the hallmarks of apoptosis and play crucial roles in this cellular process. Compared to the untreated control and the individual drugs, compound **3** plus cDDP induced a significant increase in Bax and a decrease in Bcl-2 (Fig. 4d–f).

### Compound 3 combined with cDDP inhibited phosphorylation of EGFR and downstream signaling proteins in NCI-H441 cells

**Fig. 5:**
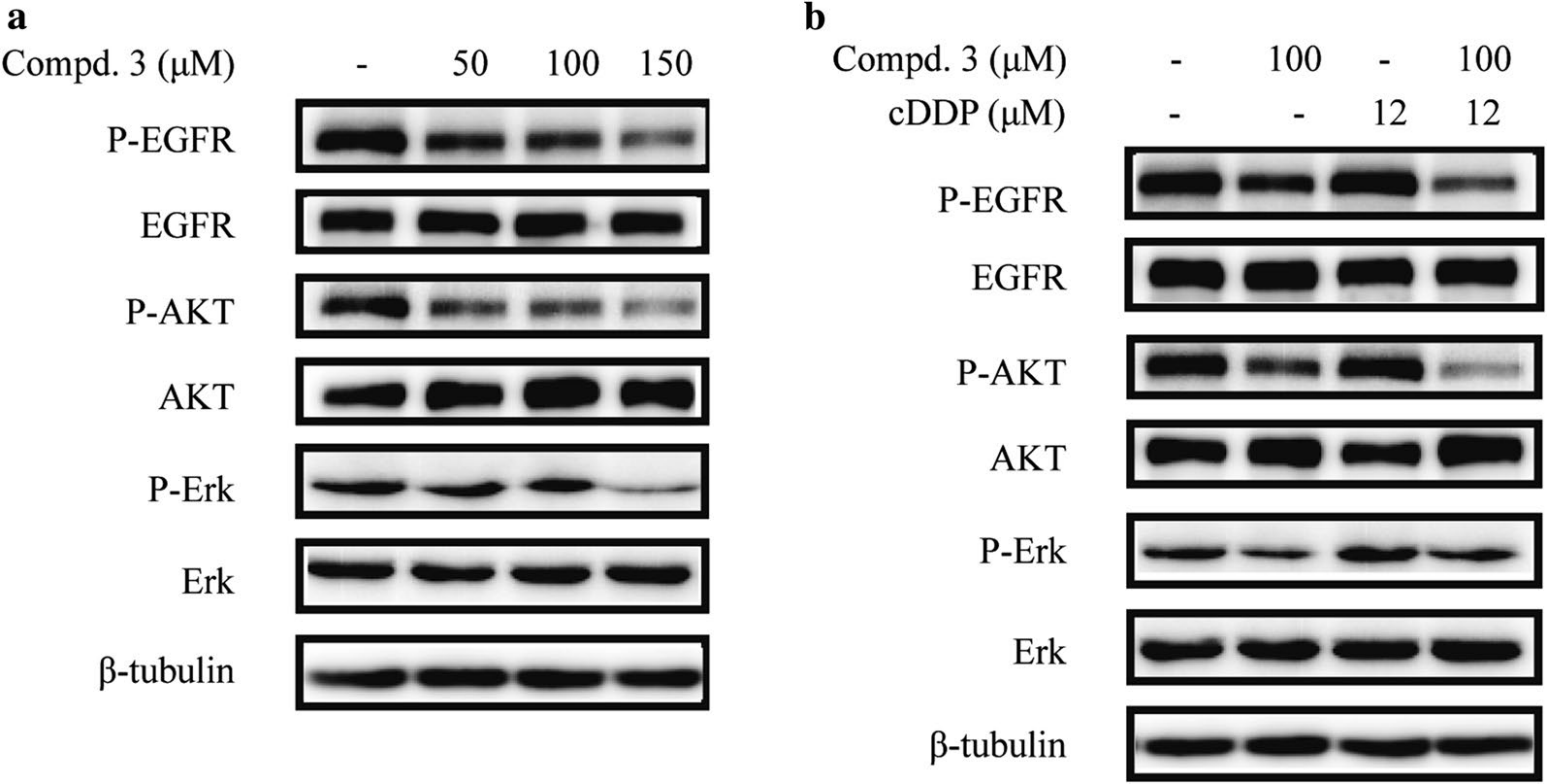
Effects of compound 3, cDDP, or a combination of the two drugs on the EGFR signaling pathway in NCI-H441 cells. **a** NCI-H441 cells treated with various concentrations of compound **3** (0, 50, 100, or 150 μM). **b** NCI-H441 cells treated with compound **3** (50 μM) and cDDP (12 μM) in combination or individually. The expression levels of the proteins were determined by western blotting after 12 h with β-tubulin as the loading control

To investigate whether the effects of compound **3** and cDDP might involve EGFR signaling, we assessed the expression of several key regulators that function within the EGFR signaling pathway. The results showed that treatment with cDDP alone slightly exhibited P-EGFR, P-AKT, and P-ERK and compound **3** alone effectively inhibited these phosphorylation proteins in a dose-dependent manner (Fig. 5a).

Moreover, compound **3** plus cDDP further inhibited the phosphorylation of EGFR and the downstream signaling proteins compared to cDDP alone, while the total protein levels of EGFR, AKT and ERK1/2 remained unchanged in each of the groups (Fig. 5b). In summary, EGFR signaling may play a significant role in the effects of the synergistic combination of compound **3** and cDDP in NCI-H441 cells.

### Transport of EGCG derivatives in Caco-2 cell monolayers

**Fig. 6:**
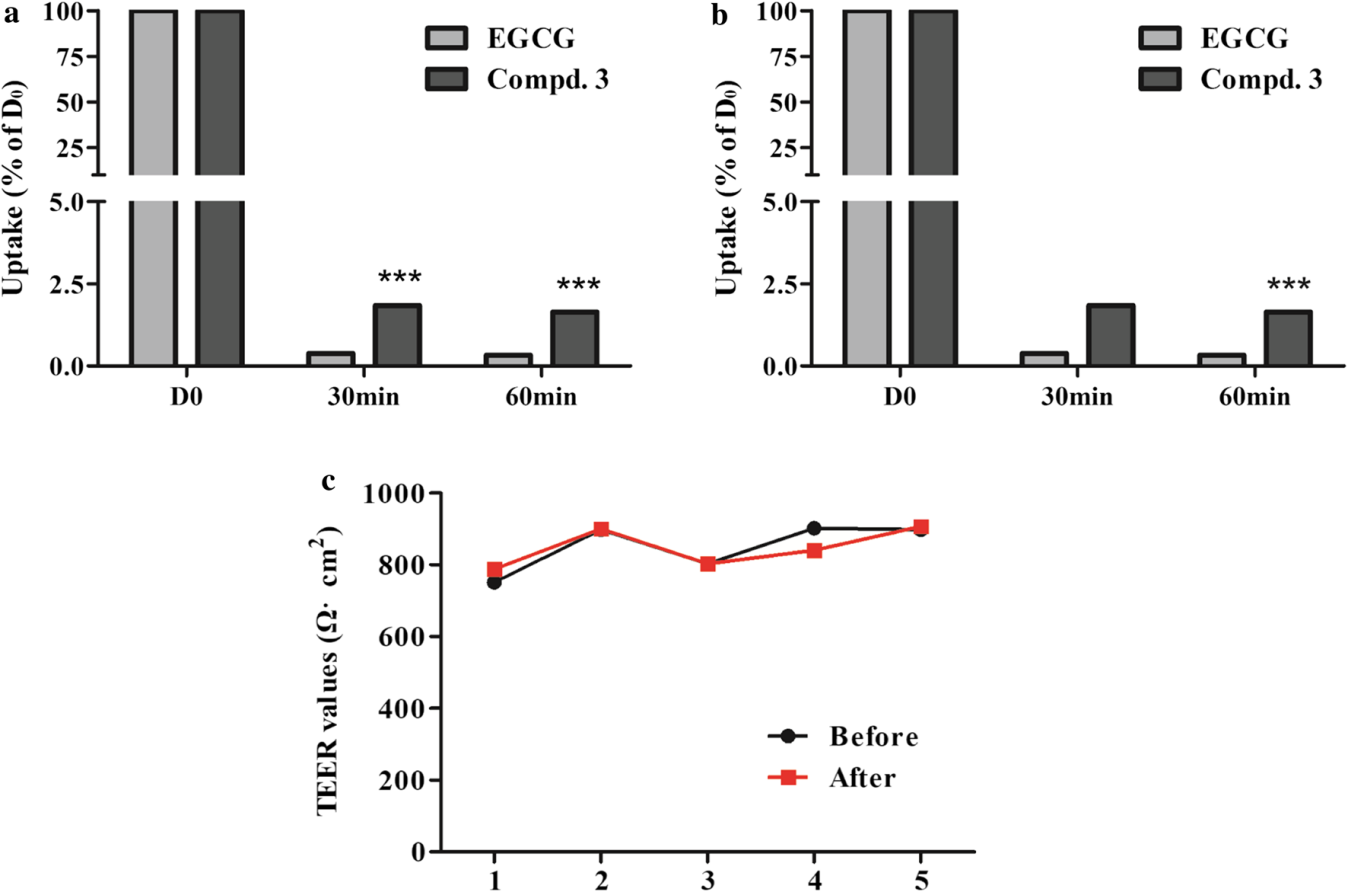
Transport of EGCG and compound 3 across Caco-2 monolayers. **a** The transepithelial transport rate of EGCG and compound **3** (both 50 μg/mL) in the apical to basolateral direction. **b** The transepithelial transport rate of EGCG and compound **3** (both 50 μg/mL) in the basolateral to apical direction. **c** TEER values of Caco-2 monolayers (Ω cm^2^). Data represent mean ± SD (*n* = 3). ***p < 0.001 compound **3** vs EGCG

The absorption of compound **3** and EGCG was studied using confluent and differentiated Caco-2 cell monolayers. As shown in Fig. 6, the uptake of compound **3** in the basolateral to apical (B→A) direction was similar to that in the apical to basolateral (A→B) direction in Caco-2 cell monolayers. Meanwhile, at a concentration of 50 µg/mL, the transepithelial transport rate of compound **3** in the apical to basolateral direction was 4.9-fold higher than the corresponding values of EGCG.

The calculated apparent permeability coefficients (*P*_app_) for EGCG and compound **3** were in the range of 0.83 × 10^−7^ to 9.14 × 10^−7^ cm/s (Table 1). These relatively small *P*_app_ values indicate that both EGCG and compound **3** showed limited transepithelial absorption, which may be associated with their low oral bioavailability. The TEER values of the monolayers did not exhibit a drop during the experimental period, indicating that the cell monolayers remained intact and that the transport of compound **3** from the apical to the basolateral chamber and vice versa did not damage the monolayers (Fig. 6c).

**Table 1:** *P*_app_ values for EGCG and compound 3 (both 50 μg/mL) from the apical to the basolateral chamber and vice versa over different times

	*P*_app_ (10^−7^ cm/s)			
	30 min (A→B)	30 min (B→A)	60 min (A→B)	60 min (B→A)
EGCG	1.89	8.9	3.2	0.83
Compound **3**	9.14	8.3	4.1	3.9

Data are reported as mean ± SD (*n* = 3)

### Effect of compound 3 in combination with cDDP on tumor growth in NCI-H441 xenograft models

**Fig. 7:**
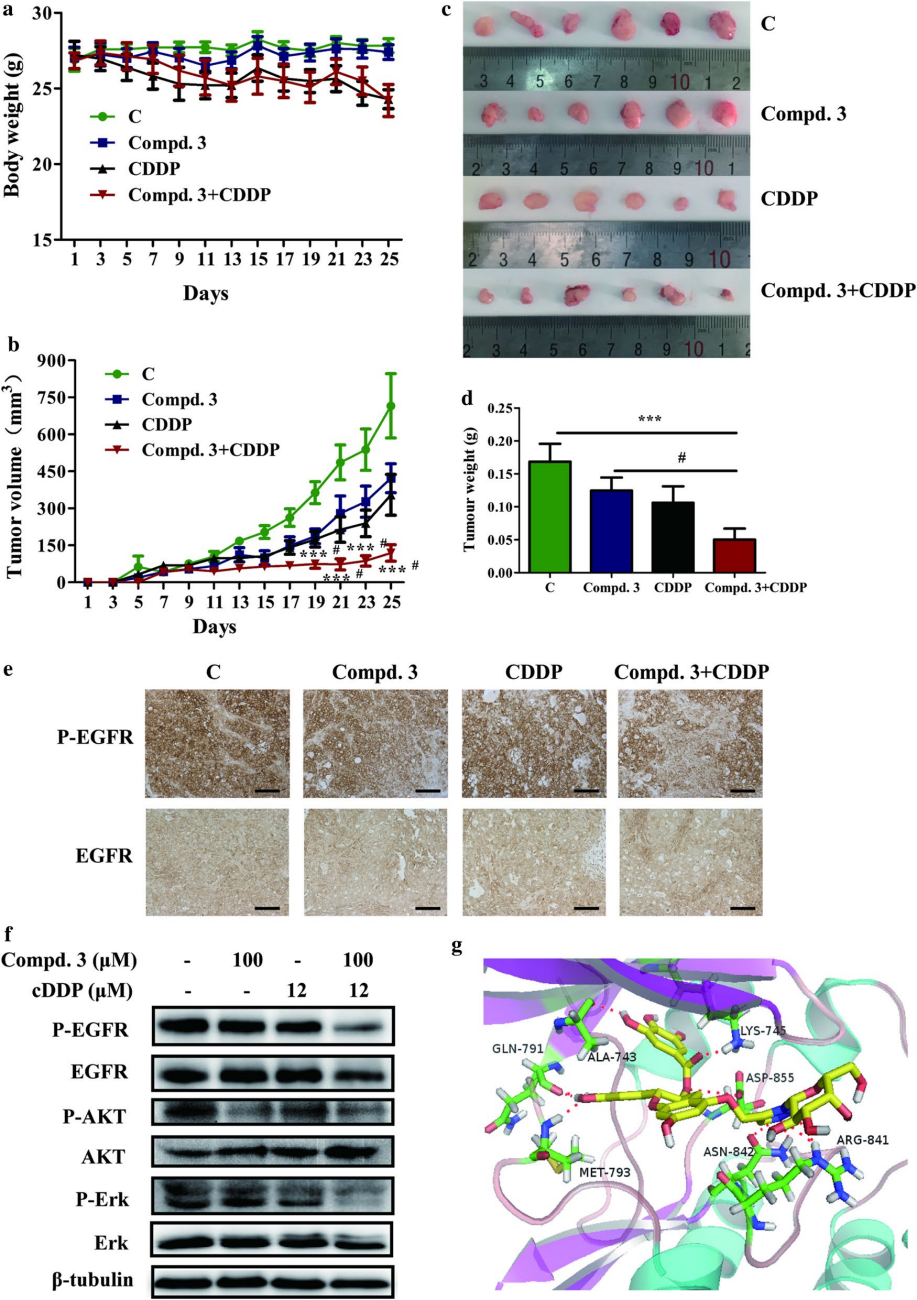
The antitumour effect of compound 3 and cDDP on NCI-H441 xenograft models. **a** The average body weight of each group. **b** The tumor volumes were measured every 2 days during the treatment. **c**, **d** The mice were sacrificed 25 days after drug treatment initiation, and the solid tumors were peeled from mouse subcutaneous tissue and tumor weights were measured. **e** Tumor tissues from NCI-H441 xenografts were immunostained with EGFR and P-EGFR antibodies. Magnification, ×20. Scale bar represents 100 μm. **f** Western blotting was used to assess EGFR, AKT, ERK, and their phosphorylated levels in tumor tissues. **g** Molecular docking model of compound **3** bound to EGFR (PDB code: 2ITY): ligand is colored by element type (C, yellow; O, red; N, blue; polar H, white), whereas key residues are shown as sticks (C, green; O, red; N, blue; polar H, white), and key interactions are denoted by a red-dotted line. Data represent the average of six independent mice per group (mean ± SD). ***p < 0.001 vs the control; ^#^p < 0.05 cDDP + compound **3** vs compound **3**

We determined the effects of compound **3**, cDDP or synergistic combination of the two drugs on tumor growth using established xenograft generated by subcutaneous dorsal implantations of NCI-H441 cells into nude mice. Consistent with the experiments in vitro, compound **3** or cDDP alone reduced tumor growth compared with the control. Furthermore, compound **3** plus cDDP significantly reduced tumor volumes and tumor weight than either single treatment (Fig. 7b–d). As shown in Fig. 7a, compound **3** treatment did not reduce the nude mouse body weight compared with the control group, which suggested that compound **3** had no apparent side effect. In agreement with the mechanistic findings in vitro, tumors in each group were analyzed by IHC staining. IHC showed that synergistic combination therapy resulted in greater inhibition of P-EGFR compared with other groups not through degradation of EGFR and the IHC for EGFR was positive (Fig. 7e). Additionally, western blot analysis of tumor tissues demonstrated that compound **3** plus cDDP resulted in greater inhibition of P-EGFR, P-AKT and P-Erk1/2 compared with the individual drugs and control groups (Fig. 7f).

### Molecular docking study

To understand the interaction between compound and kinases, the possible binding modes of compound **3** on EGFR (PDB code: 2ITY) was explored using the AutoDock 4.2. As shown in Fig. 7g, the nitrogen atoms of the 1,2,3-triazole ring forms hydrogen bond with the amide hydrogen atom of ASN-842 and ARG-841. The carbonyl oxygen atom of compound **3** also forms hydrogen bond with the amide hydrogen atom of LYS-745. Besides, the interactions of C3′-OH and C4′-OH in the side chain with MET-793 and GLN-791 and C3″-OH with ALA-743. A much more detailed molecular biological study elucidating this chelated binding and the mechanism of action is ongoing in our laboratory and will be reported in due course.

## Discussion

Lung cancer is the leading cause of cancer-related mortality worldwide and several efficient antibodies and drugs have been developed for its treatment [20]. Platinum-based chemotherapy, such as cDDP, is a conventional treatment for most advanced NSCLC patients, but therapeutic resistance is a major obstacle. To overcome such resistance, the exploration and development of novel compounds are urgently needed [21]. Compounds from natural sources represent an indispensable candidate drug library for pharmacotherapy. In this study, we studied the antitumor effects of compound **3**, including the growth inhibition of NSCLC cells, suppression of colony formation, induction of apoptosis, and inhibition of signaling pathways.

In a previous study it was reported that EGCG increases the levels of miR-210 in human and mouse lung cancer cells, resulting in a significant reduction in proliferation and anchorage-independent growth [22]. Furthermore, EGCG inhibited the growth of lung cancer cells in a dose-dependent manner in cell cultures and xenograft tumors [23]. Other studies have demonstrated that EGCG enhanced the sensitivity of lung cancer cells to cDDP [24]. We therefore investigated whether EGCG derivatives inhibit the growth of lung cancer cells as well as EGCG. The results demonstrated that compound **3** inhibited the cell viability and colony formation for NCI-H441 (Fig. 2). In the current study, cDDP and compound **3** were found to have a synergistic inhibitory effect on the growth of NCI-H441 cells when used in combination, whereas the two compounds only exhibited slight inhibitory effects on lung cancer growth when used individually.

The effects of compound **3** with or without cDDP on NCI-H441 cell apoptosis were also investigated using annexin V-FITC and PI fluorescence staining. It was notable that the compound **3** plus cDDP led to a significantly increased expression ratio of Bax/Bcl-2, which further supports that compound **3** plus cDDP was capable of inducing apoptosis in NCI-H441 cells and that compound **3** induces the apoptosis via Bcl-2 and Bax modulation. Similarly, Wang et al. reported that the combination of gefitinib and gambogic acid induces Bax and Bcl-2 mediated apoptotic cell death in NSCLC cells [25].

The EGFR signaling pathways play a critical role in proliferation, invasion, and survival in NSCLC [26]. PI3K/Akt and RAS/RAF/MEK/ERK are two main downstream pathways of EGFR signaling [27, 28]. In our study, EGFR, Akt, ERK, and their phosphorylated forms were selected for examination as potential mediators of cDDP and compound **3** signaling through the EGFR in NSCLC. In NCI-H441 cells, compound **3** alone can inhibit the EGFR signal pathway, although compound **3** treated with cDDP led to increased inhibition due to synergistic effects.

The prevalence of KRAS mutations in the lung cancer is 20% to 30%, 85% of which affect codon 12, and these mutations are associated with poor prognosis in NSCLC patients [29]. The interaction test for chemotherapy benefit and KRAS mutational status was not significant [30]. Despite this KRAS mutation has been shown to be associated with poor prognosis, no clinical significance was reported, since no difference in the response to chemotherapy treatment between patients harbouring codon 12 mutated -KRAS or wild-type KRAS was found [31, 32]. Data do not support the routine use of KRAS mutational analysis to predict chemotherapy benefit. There had been significant interest in using KRAS status to select patients for EGFR TKI and anti-EGFR monoclonal antibodies [31].

There are still some limitations of our study. The transepithelial transport rate of compound 3 was higher than EGCG in Caco-2 cell monolayers. However, we did not investigate the absorption and stability of EGCG and compound 3 in vivo, we will demonstrated if compound 3 is more stable also in vivo. Besides, we did not investigate the mechanism of A549 cells with wild type EGFR are more sensitive to compound 3 treatment compared with that of NCI-H1975 cells with EGFR L858R/T790M mutation. Future studies will determine the role of EGFR status in the response to EGCG derivatives. Nevertheless, our study provide the foundations for further work about potential natural compound for patients.

The catechins found in green tea have received considerable attention due to their favorable bioactivity. However, their therapeutic potential still remains limited by their low oral bioavailability, which is attributable to poor stability and intestinal absorption [33]. In this study, several new derivatives were developed to enhance the stability of EGCG. Compared with EGCG, the EGCG derivatives displayed enhanced transport in Caco-2 cell monolayers. It provides new research mentality for the development and application of natural compounds.

## Conclusions

In conclusion, we have shown that cDDP combined with compound **3** promotes anti-proliferation, cell cycle redistribution, apoptosis, and inhibition of the EGFR signaling pathway in NCI-H441 cells. Furthermore, combination treatment with compound **3** and cDDP exhibited synergistic effects on suppresses tumor growth in vivo. In particular, compound **3** has the potential in NSCLC treatment.

## Data Availability

The datasets and material used and analyzed during the current study are available from the corresponding author upon request.

## Abbreviations

cDDP: cisplatin
EGFR: epidermal growth factor receptor
EGCG: (−)-epigallocatechin-3-gallate
compd: compound
AKT: protein kinase B
ERK: extracellular regulated protein kinases
CDK2: cyclin-dependent kinase 2
PCNA: proliferating cell nuclear antigen
SDS: sodium dodecyl sulfate
PVDF: poly(vinylidene fluoride)
PARP: poly-ADP-ribose polymerase
